# Mechanically Reprogrammed Coaxial Fibers for Multiphysics Transduction and Human‐Machine Interfaces

**DOI:** 10.1002/advs.77580

**Published:** 2026-09-06

**Authors:** Xinyi Cao, Chao Ye, Shengjie Ling, Ming Li, Zhao Sha, Chun‐Hui Wang, Shuhua Peng

**Affiliations:** ^1^ School of Mechanical and Manufacturing Engineering University of New South Wales Sydney NSW Australia; ^2^ School of Textile and Clothing Yancheng Institute of Technology Yancheng Jiangsu China; ^3^ State Key Laboratory of Molecular Engineering of Polymers Department of Macromolecular Science Fudan University Shanghai China; ^4^ Centre for Advanced Manufacturing Technology School of Engineering Faculty of Engineering Computing and Science Western Sydney University Penrith NSW Australia

**Keywords:** coaxial wet spinning, human‐machine interfaces, mechanical reprogramming, self‐powered sensing

## Abstract

Fiber‐shaped electronic systems provide a promising platform for wearable sensing and intelligent textiles, yet the continuous processing of uncured silicone‐based conductive materials remains challenging because of their low structural stability during fiber formation. Here, we develop a coaxial wet‐spinning strategy that enables the continuous aqueous‐bath confinement of an uncured PDMS/CNT conductive core. During spinning, a rapidly phase‐separating PVDF‐HFP sheath provides immediate radial confinement, preventing leakage and structural collapse of the hydrophobic liquid core, while a UV‐crosslinked PEGDA secondary network reinforces the sheath after fiber formation. Unlike removable templates or sacrificial supporting layers, the PVDF‐HFP/PEGDA sheath is permanently retained as the dielectric and load‐bearing component, thereby directly forming an integrated dielectric–electrode coaxial fiber. The resulting PVDF‐HFP/PEGDA@PDMS/CNT fibers can be continuously fabricated at the meter scale and exhibit reversible sheath microstructural evolution, stable strain‐dependent resistance, and reliable performance under repeated deformation. The integrated dielectric–electrode architecture also allows an individual fiber to generate triboelectric signals without additional device assembly. This work demonstrates a practical route for continuously processing uncured PDMS‐based conductive materials in an aqueous coagulation bath and integrating mechanical, resistive, and triboelectric functions within a permanently retained coaxial fiber architecture.

## Graphical abstract

A continuously wet‐spun coaxial fiber is developed to combine stretchable conductivity, resistive sensing, and self‐powered triboelectric sensing in a single architecture. A mechanically reprogrammed dual‐network sheath stabilizes large deformation and improves signal reliability. These fibers can be woven into textile interfaces for multichannel touch sensing, interactive control, and wearable human–machine interaction.

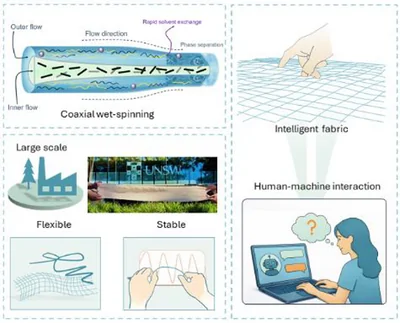

## 1 Introduction

Fiber‐based electronics are increasingly recognized as a promising platform for wearable sensing, intelligent textiles, and human–machine interfaces due to their intrinsic flexibility, weavability, and geometric scalability [1, 2, 3, 4, 5]. From a manufacturing perspective, wet spinning has long served as a foundational and scalable strategy for the continuous production of functional fibers [6, 7, 8]. In conventional aqueous wet spinning, the solidification of the spinning dope typically relies on rapid phase separation or chemical crosslinking within the coagulation bath [9, 10].

However, extending aqueous wet spinning to silicone‐based elastomers remains challenging. Among various material systems, polydimethylsiloxane (PDMS)‐based conductive composites incorporating carbon nanotubes or liquid metals have demonstrated outstanding performance in flexible sensing, soft electronics, and self‐powered systems [5, 11, 12, 13]. Despite these advantages, PDMS behaves fundamentally differently from polar polymers in aqueous environments [14, 15, 16, 17]. It neither undergoes phase separation nor stabilize in water, resulting in jet instability and structural collapse during wet spinning [18, 19]. Consequently, the continuous fabrication of PDMS‐based conductive fibers via conventional wet spinning remains a significant challenge.

To overcome this limitation, several alternative fabrication strategies have been explored. Reported approaches for fabricating PDMS‐based fibers generally rely on external geometrical confinement or rapid in situ crosslinking, including template‐assisted molding [20], extrusion or drawing coupled with thermal curing [21], and thermally induced oil‐bath spinning [22, 23, 24]. Although these approaches enable the formation of PDMS‐based elastomeric filaments, stabilization of the initially fluid precursor generally relies on predefined geometrical confinement, rapid thermal crosslinking, heated nonaqueous baths, or subsequent removal of a temporary supporting structure. These requirements can increase processing complexity and make it difficult to simultaneously achieve continuous fiber formation and direct integration of functional core–sheath components [25, 26]. As a result, elastomeric conductors are often restricted to extrusion‐based or post‐infiltration fabrication routes, which remain difficult to scale and poorly compatible with textile‐level fiber production.

Coaxial wet spinning offers a promising strategy to address these challenges by constructing multiphase core–sheath architectures. In such systems, a sheath layer that can rapidly solidify in the coagulation bath provides temporary structural support for a soft or non‐coagulable core, such as PDMS‐based elastomeric conductors. This structural design enables the continuous fabrication of fibers containing materials that cannot independently stabilize during aqueous wet spinning. As a result, coaxial spinning has been widely explored for producing stretchable conductive fibers and elastomer‐based composite filaments with improved structural integrity [27, 28, 29]. In commonly used designs, polar polymers such as PVDF and its copolymers serve as protective sheath materials [30, 31]. Under cyclic tensile loadings, these polymers often exhibit mechanical hysteresis and irreversible structural rearrangement, leading to electrical signal drift and functional instability [29, 32]. Consequently, although coaxial wet spinning improves fabrication compatibility, it does not fully resolve the issue of mechanical stability during long‐term service. Moreover, considerable efforts have been devoted to stretchable conductive fibers and fiber‐shaped triboelectric nanogenerators (TENGs) [33, 34], enabling promising demonstrations in self‐powered sensing and textile electronics [35, 36, 37]. Nevertheless, most existing systems rely on separate fabrication routes for conductive fibers, energy harvesters, and interactive modules, resulting in fragmented manufacturing strategies [38, 39, 40].

**FIGURE 1:**
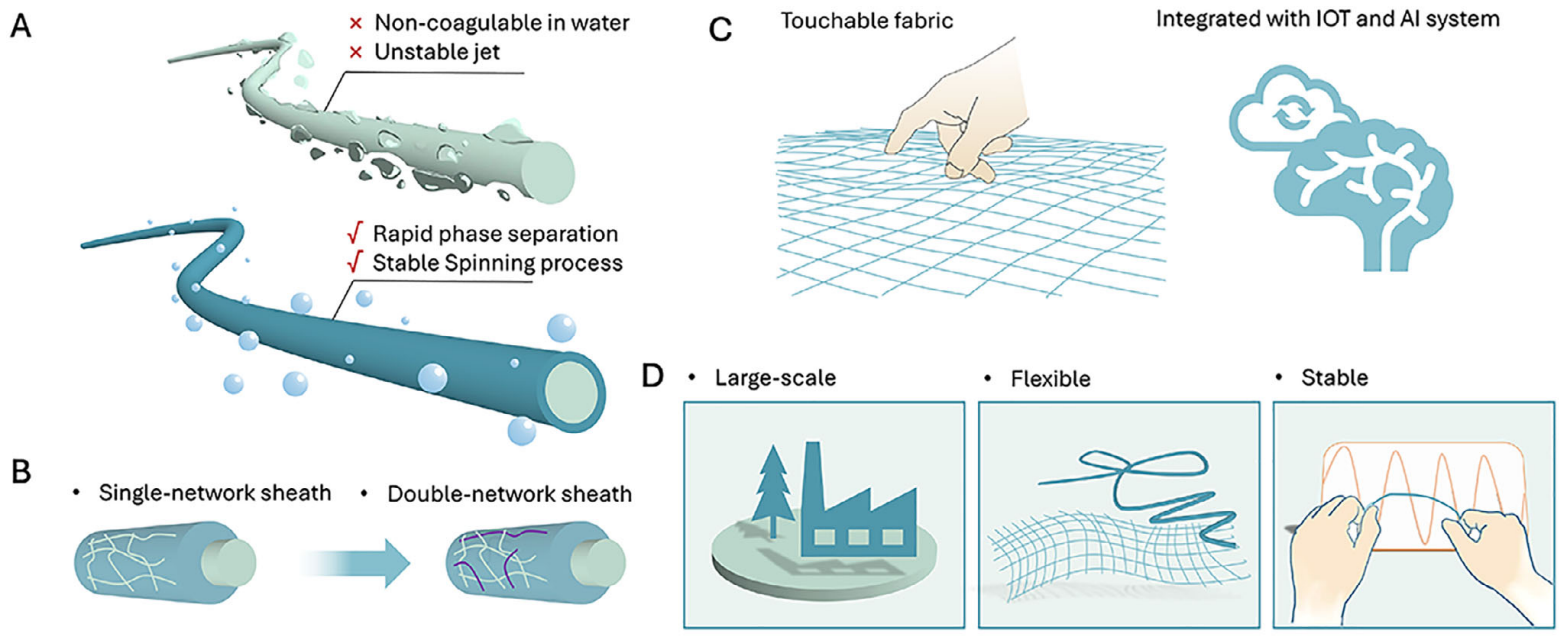
Design principle and structural concept of the dual‐network coaxial fiber platform.

To address this limitation, we propose a fiber‐level structural strategy that decouples geometric stabilization from material solidification during aqueous bath wet spinning, enabling continuous fabrication of PDMS‐based conductive fibers with improved mechanical reliability. A rapidly phase‐separating polar sheath transiently stabilizes the conductive PDMS core during fiber formation, after which the confined precursor is cured to yield well‐defined core–shell fibers that preserve elastomeric compliance [30, 41, 42]. A dual‐network sheath further redistributes strain and suppresses localized yielding, enabling reversible deformation and stable electrical performance under repeated loading. The resulting coaxial fibers combine meter‐scalable manufacturability, flexibility, and long‐term stability, while the integrated conductive–dielectric architecture allows direct operation as self‐powered triboelectric sensors. When woven into textiles and coupled with Internet of Things (IoT) modules and lightweight AI algorithms, the system enables real‐time signal transmission, tactile‐input decoding, and human–machine interaction, establishing a pathway from meter‐scalable fiber manufacturing to intelligent textile systems. Figure 1 illustrates the design principles and structural concept underlying the dual‐network coaxial fiber platform in this work.

## 2 Results and Discussion

### 2.1 Meter‐Scalable Aqueous‐Bath Coaxial Wet Spinning

**FIGURE 2:**
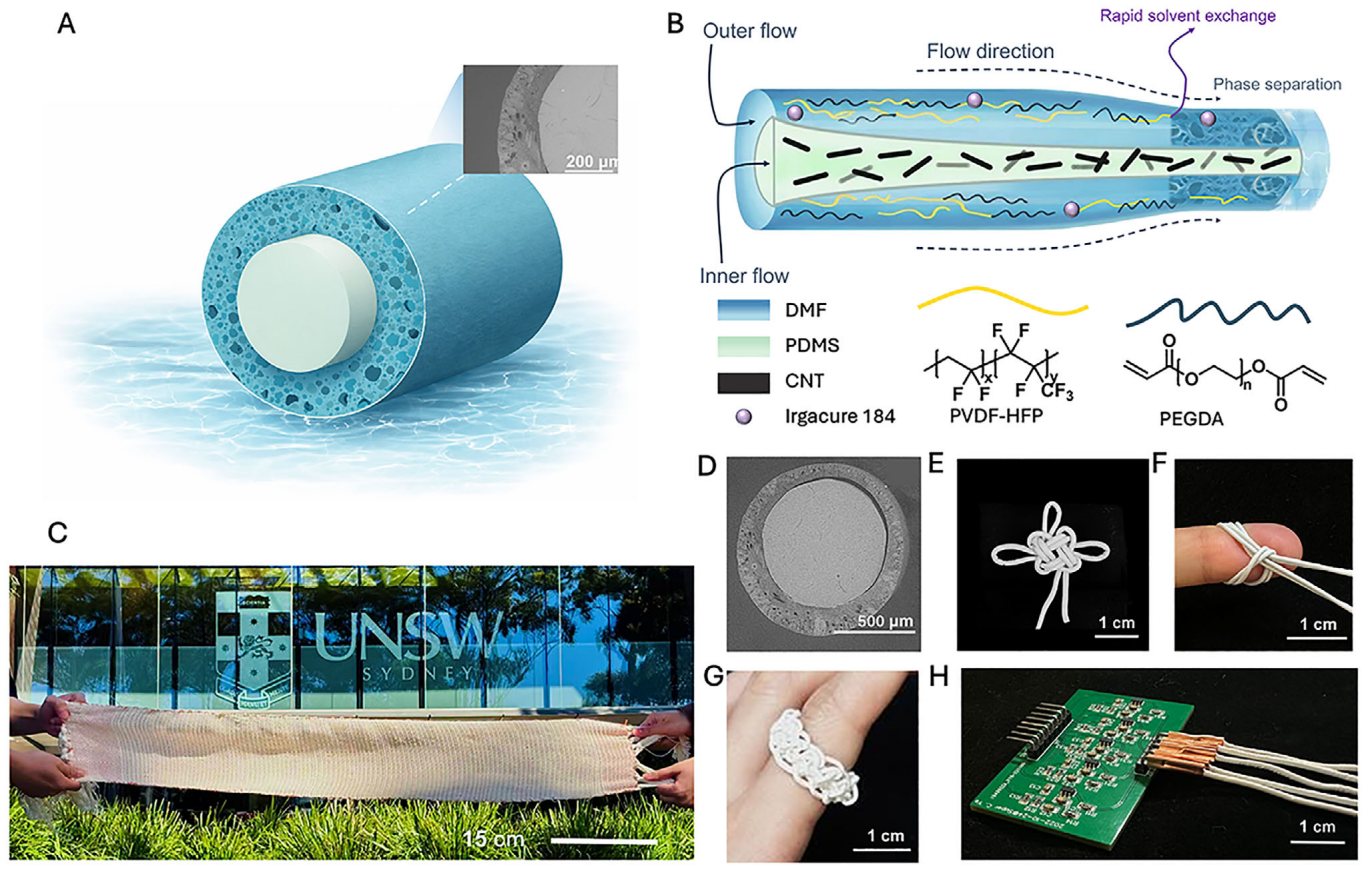
Fabrication and structural characterization of the D^2^CFs. (A) Schematic illustration and cross‐sectional morphology of the coaxial fiber formed in a water coagulation bath. (B) Working mechanism of the coaxial wet‐spinning process, showing rapid solvent exchange, phase separation of the PVDF‐HFP sheath, and confined extrusion of the PDMS/CNT core. (C) Photograph of the continuously wet‐spun fiber at the meter scale. (D) SEM cross‐sectional image of the dual‐network coaxial fiber. (E) Demonstration of mechanical flexibility via a Chinese‐knot configuration. (F) Mechanical flexibility demonstrations, including knotting and bending. (G) Wearable demonstration of a simple loop structure mounted on a finger. (H) 3D woven configuration integrated around a finger. (I) Electrical connection of multiple fibers integrated onto a circuit board.

Guided by a single coaxial extrusion process into an aqueous coagulation bath, enabling the continuous fabrication of PVDF‐HFP/PEGDA@PDMS/CNT dual‐network coaxial functional fibers (D^2^CFs) (Figure 2A). The core of this process lies in preserving the conventional aqueous wet‐spinning framework while introducing instantaneous geometric confinement through rapid sheath phase separation. In this way, the non‐coagulable elastomeric conductor of PDMS/CNT can be stabilized during the critical jet‐formation stage without modifying its intrinsic curing chemistry (Figure 2B). Specifically, a PVDF‐HFP/PEGDA solution in DMF was employed as the sheath spinning dope. Upon extrusion into a deionized water coagulation bath, rapid solvent exchange and phase separation occur, forming a continuous outer shell within a millisecond‐to‐second timescale [43, 44, 45]. Simultaneously, the PDMS/CNT prepolymer was extruded through the inner channel of the coaxial spinneret. Importantly, the PDMS/CNT system does not undergo coagulation or significant viscosity change in water; direct exposure to the aqueous bath would lead to jet breakup and leakage. However, under the radial confinement established by the rapidly solidified sheath, the uncured PDMS/CNT core remains axially continuous and can be stably drawn. This dynamic confinement enables the continuous formation of the coaxial core–sheath structure through a single coaxial extrusion step in an aqueous coagulation bath, effectively overcoming the inherent incompatibility between elastomeric conductors and wet spinning.

In the optimized spinning formulation, the sheath solution consisted of 20 wt.% PVDF‐HFP in DMF, with PEGDA and the photoinitiator Irgacure 184 added at 4 and 2 wt.% relative to the PVDF‐HFP mass, respectively. The core was composed of a PDMS/CNT composite, with a CNT content of 2.5 wt.%. During coaxial wet spinning, the extrusion rates of the sheath and core were maintained at 10 and 12 mL h^−^^1^, respectively. Within this parameter window, the coaxial jet exhibited stable propagation in the aqueous coagulation bath, with no observable interfacial rupture, jet oscillation, or core leakage. This stability confirms that rapid sheath solidification provides sufficient mechanical scaffolding during the early stage of fiber formation. After initial shape fixation in the water bath, the fibers were further treated in deionized water at 70°C for 4 h to complete crosslinking of the PDMS core. Subsequently, the dried fibers were exposed to 365 nm UV irradiation for 10 min to initiate PEGDA network formation within the sheath. Figure S1 presents the FTIR spectra of pure PVDF, the PVDF/PEGDA system before photoinitiated crosslinking, and the PVDF/PEGDA network after UV curing. Compared with pure PVDF, both PVDF/PEGDA samples exhibit a characteristic ester carbonyl stretching band at approximately 1720 cm^−^^1^, demonstrating that PEGDA was retained in the PVDF matrix after aqueous bath processing. In the uncured PVDF/PEGDA system, an additional absorption peak at approximately 1630 cm^−^^1^ is assigned to the C═C stretching vibration of the acrylate groups in PEGDA. Following UV irradiation, this peak disappears while the carbonyl band remains, indicating the consumption of the acrylate double bonds and confirming the in situ photopolymerization of PEGDA to form a secondary network within the PVDF matrix. This sequential curing strategy embodies the “formation‐first, solidification‐later” decoupling principle: geometric stabilization during spinning is temporally separated from network crosslinking, allowing independent optimization of processability and final mechanical–electrical performance. By avoiding premature curing during jet formation, the effective processing window is significantly broadened.

Owing to the rapid structural confinement provided by the sheath during coagulation, the coaxial wet‐spinning system exhibits robust macroscopic continuity and scalability. Under the present laboratory conditions, fibers can be continuously produced to lengths exceeding 15 m without fracture, jet instability, or structural collapse (Figure 2C). Notably, the attainable fiber length in this study is primarily constrained by laboratory‐scale collection and spatial limitations rather than intrinsic material or process instability. Since the spinning process fully operates within a conventional aqueous bath wet‐spinning framework, the strategy is inherently compatible with continuous fiber manufacturing platforms.

The as‐spun D^2^CFs exhibit a well‐defined and uniform core–sheath architecture. Cross‐sectional SEM images (Figure 2D) reveal a continuous PVDF‐based outer sheath and a clearly delineated PDMS/CNT inner core. The overall fiber diameter is approximately 1.0 mm (±110 µm), with an average sheath thickness of ∼137 µm and a core diameter of ∼836 µm. Owing to rapid solvent exchange and phase separation in the aqueous bath, the sheath displays a porous microstructure, whereas the PDMS core remains dense and elastomerically continuous. Higher‐magnification SEM observations (Figure S2) confirm uniform dispersion of CNTs within the PDMS matrix, forming a percolated conductive network without evident aggregation or phase segregation. Such homogeneous distribution is critical for ensuring stable electromechanical coupling without compromising mechanical compliance. Raman spectroscopy of the PDMS/CNT core (Figure S3) further verifies the structural integrity of the carbon network. Both pristine CNT and PDMS/CNT samples exhibit characteristic D (∼1330 cm^−^^1^) and G (∼1580 cm^−^^1^) bands associated with defect‐induced disorder and in‐plane sp^2^ carbon stretching vibrations, respectively. The negligible shift in peak position and preserved peak sharpness after composite formation indicate that the incorporation of PDMS does not disrupt the sp^2^ carbon framework. This preservation of graphitic structure is essential for maintaining reliable strain‐sensitive conduction and triboelectric signal generation. The “sheath‐first formation, core‐later curing” evolution pathway therefore establishes both morphological integrity and functional readiness, laying the foundation for stable strain‐resistive response and long‐term electrical reliability.

Beyond microstructural integrity, the D^2^CFs demonstrate excellent macroscopic processability and mechanical compliance. The fibers can be freely knotted, coiled, and woven without preconditioning, maintaining structural continuity under repeated bending and high‐curvature deformation. As illustrated in Figure 2E, the fiber preserves its integrity within a Chinese‐knot configuration featuring a local curvature radius as small as ∼1.5 mm, indicating robust resistance to bending‐induced fracture. Owing to their geometric stability and mechanical resilience, the fibers can be directly embroidered into conventional textile substrates without structural reinforcement (Figure S4). Once integrated, the fibers remain mechanically anchored within the woven matrix, demonstrating compatibility with established textile fabrication techniques. Individual fibers can further be configured into simple looped geometries or more complex 3D wearable structures and conformally mounted onto the human body, such as around a finger (Figure 2F,G). These demonstrations confirm that the coaxial architecture sustains mechanical integrity across diverse deformation modes encountered in different wearable scenarios. Importantly, the fibers establish stable electrical contact with standard electronic terminals (Figure 2H), evidencing reliable interface compatibility between soft textile platforms and rigid semiconductor components. This electrical interconnect stability underscores the system‐level viability of the proposed fiber architecture for integration into existing electronic infrastructures.

### 2.2 Mechanical Reprogramming of the Dual‐Network Sheath

After the successful fabrication of elastomeric conductive fibers via aqueous bath coaxial wet spinning, long‐term stability during service is governed by how strain is accommodated within the sheath layer. Irreversible yielding of the sheath under tensile loading can generate stress concentration at the core–sheath interface, which may propagate into the compliant conductive core and cause electrical drift or signal instability. To regulate this strain accommodation pathway, a secondary network was introduced into the PVDF‐based sheath to mechanically reprogram the overall fiber response.

**FIGURE 3:**
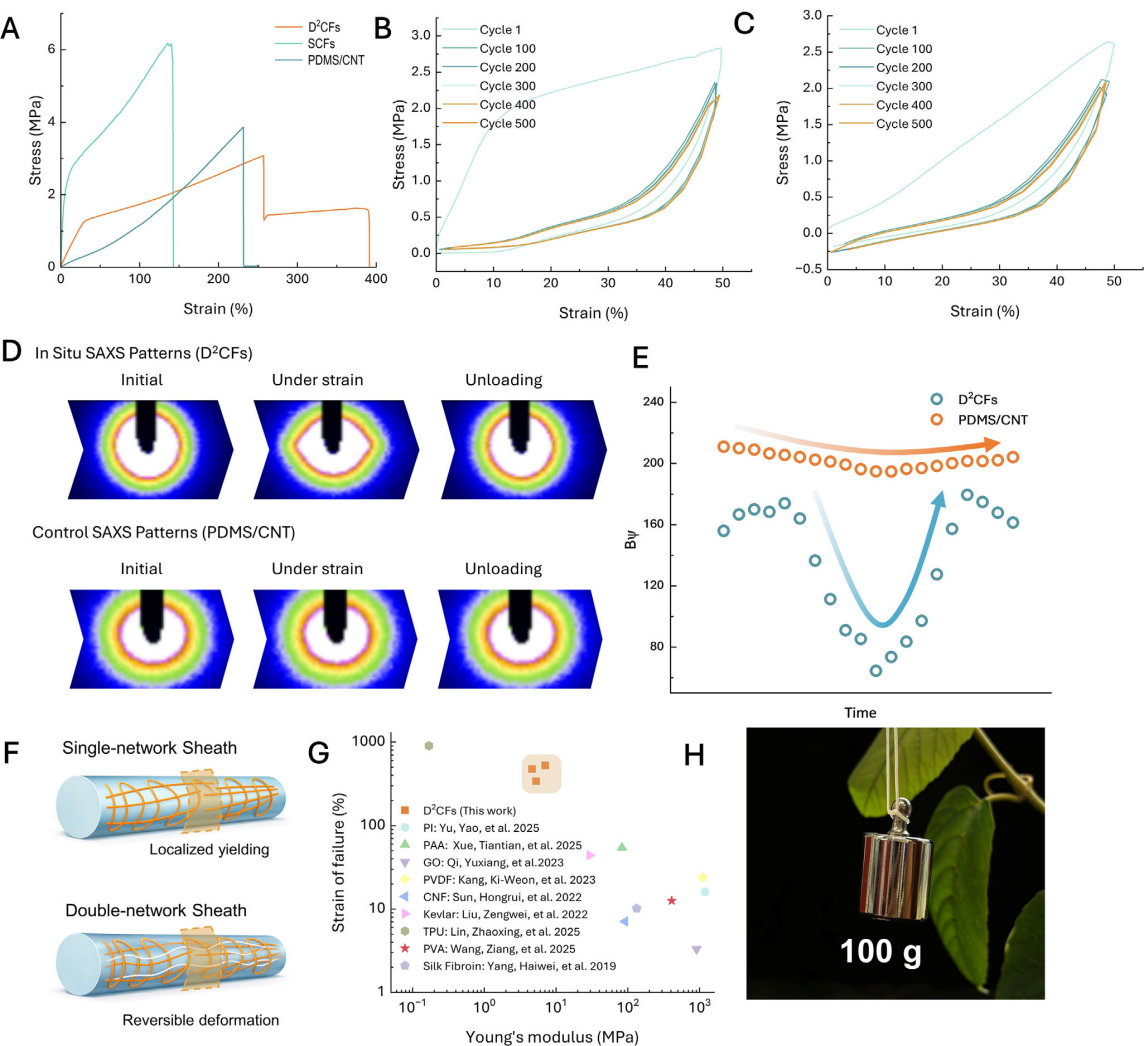
Mechanical reprogramming and structural evolution of D^2^CFs. (A) Stress–strain curves of D^2^CFs, SCFs, and PDMS/CNT core materials. (B) Cyclic tensile behavior of SCFs under repeated loading. (C) Cyclic tensile behavior of D^2^CFs under repeated loading. (D) In situ SAXS patterns of D^2^CFs during a loading–unloading cycle, with corresponding control SAXS patterns of PDMS/CNT. (E) Evolution of the orientation parameter *B*_Ψ_ during cyclic deformation for D^2^CFs and PDMS/CNT. (F) Schematic comparison of deformation mechanisms in single‐network and dual‐network sheaths, illustrating localized yielding versus reversible deformation. (G) Comparison of Young's modulus and strain‐to‐failure between D^2^CFs and representative porous fiber systems reported in the literature. (H) Load‐bearing demonstration of a single D^2^CF supporting a 100 g weight.

To elucidate the necessity of the dual‐network sheath, the mechanical behavior of individual components was first characterized independently. The standalone PDMS/CNT elastomeric conductor exhibits typical rubber‐like tensile behavior, with a fracture strain of approximately 230% (Figure 3A). After 500 loading–unloading cycles at 0% pre‐strain, minimal mechanical hysteresis is observed (Figure S5). Furthermore, during stepwise loading–unloading from 20% to 200% strain, the stress–strain curves largely overlap (Figure S6), indicating good elastic recovery and limited irreversible deformation. These results confirm that the PDMS/CNT core itself is not the limiting factor governing overall fiber stability.

In contrast, when the sheath consists solely of a single PVDF network (single‐network coaxial functional fibers, SCFs), the mechanical deformation of the coaxial fiber is strongly constrained by the relatively rigid sheath. The SCFs exhibit a fracture strain of only approximately 130%, which is substantially lower than the intrinsic stretchability of the PDMS/CNT core (Figure 3A). Statistical tensile measurements from multiple fiber specimens further show that the SCFs possess a higher Young's modulus and a lower fracture strain than the dual‐network coaxial functional fibers (D^2^CFs) (Figure S7). This mechanical mismatch between the single‐network PVDF sheath and the soft PDMS/CNT core limits the usable strain range of the coaxial fiber.

After introducing PEGDA as a secondary network into the PVDF sheath, both the compliance and stretchability of the coaxial fiber are significantly improved. The D^2^CFs exhibit a lower Young's modulus and a fracture strain approaching 400%, as confirmed by the statistical results obtained from multiple fiber specimens (Figure S7). During tensile loading, the PDMS/CNT core fractures first, resulting in the initial stress drop observed in the stress–strain curve, while the PVDF/PEGDA sheath remains intact and continues to sustain further deformation until its subsequent fracture (Figure 3A). This sequential failure behavior delays the complete fracture of the coaxial fiber and demonstrates the ability of the dual‐network sheath to maintain the structural integrity of the fiber after core failure. The combination of a lower Young's modulus and a higher fracture strain provides the D^2^CFs with improved deformability and mechanical compatibility for wearable textile applications.

The cyclic mechanical behaviors of the SCFs and D^2^CFs were further evaluated through loading–unloading tests at a fixed strain of 50% for 500 cycles. Both fibers exhibit hysteresis and stress softening, particularly during the initial loading cycles, followed by a gradual stabilization of their mechanical responses (Figure 3B,C). However, the D^2^CFs show a smaller first‐cycle hysteresis than the SCFs, as quantitatively summarized in Figure S8, indicating reduced irreversible structural rearrangement and energy dissipation during the initial deformation. After 500 cycles, the D^2^CFs retain 88.94% of their initial maximum stress, whereas the SCFs retain only 73.76%, demonstrating that the PEGDA secondary network effectively mitigates cyclic stress softening and improves long‐term mechanical stability.

The difference between the two fibers is also evident during stepwise loading–unloading tests with progressively increasing strain. Although the hysteresis increases with applied strain for both fibers, the D^2^CFs consistently exhibit smaller hysteresis loops than the SCFs over the investigated strain range (Figures S9 and S10). This behavior indicates that the dual‐network sheath can accommodate increasing deformation with a larger recoverable component and reduced irreversible energy dissipation. Therefore, the improved mechanical performance of the D^2^CFs does not arise from increased sheath stiffness, but from more effective strain redistribution and the suppression of irreversible deformation within the PVDF/PEGDA sheath. Collectively, these results demonstrate that the secondary PEGDA network reprograms the PVDF sheath from a relatively stiff and deformation‐constraining structure into a more compliant, strain‐accommodating, and cyclically stable network through improved strain redistribution and reduced irreversible deformation.

To probe the structural origin of the mechanical reprogramming, in situ synchrotron SAXS measurements were performed on the dual‐network coaxial fibers during tensile–release deformation (Figure 3D). Representative patterns corresponding to the initial state, maximum strain, and full recovery are shown in Figure 3D, while the complete sequence is provided in Figure S11. Under cyclic tensile strain (60%), the initially near‐isotropic scattering pattern gradually evolves into a pronounced elliptical shape, indicating increasing molecular orientation within the PVDF/PEGDA sheath. Upon unloading, the scattering pattern largely recovers to its original isotropic state, demonstrating the high reversibility of the sheath microstructure. For comparison, in situ SAXS measurements were performed on the standalone PDMS/CNT composite under identical strain conditions. The scattering patterns show negligible evolution (Figure 3D), indicating that the conductive core contributes minimally to the observed SAXS response and confirming that the reversible structural changes primarily originate from the dual‐network sheath. To quantitatively evaluate the orientation evolution during tensile–release deformation, the 2D SAXS patterns were azimuthally integrated and analyzed using the Ruland method. Detailed calculation procedures are provided in Note S1.

In brief, within the selected scattering vector range (q = 3.70 × 10^−^^2^ to 3.49 × 10^−^^1^), azimuthal intensity distributions were analyzed at multiple outer q positions. The observed azimuthal broadening parameter, *B_obs_* (s), was then extracted. Subsequently, the data were fitted according to the following linear relationship:

(1) $$sB_{obs}\;\left(s\right)=\;sB_{\Psi }+\frac{1}{L}$$

Here, s denotes the scattering vector used in the Ruland analysis and corresponds to the selected q‐range. *B*_Ψ_ represents the broadening contribution arising from orientation distribution (corresponding to the slope of the linear fit), while *L* denotes the effective correlation length (reciprocal of the intercept). Accordingly, *B*_Ψ_ serves as a quantitative descriptor of the angular distribution width of scattering entities: a smaller *B*_Ψ_ value indicates a narrower orientation distribution and a higher degree of axial ordering. Figure 3E presents the evolution of *B*_Ψ_ during a complete tensile–release cycle as a function of strain (or time). For the D^2^CFs, *B*_Ψ_ decreases markedly with increasing strain during loading and reaches a minimum at maximum elongation. Upon unloading, *B*_Ψ_ gradually returns toward its initial value, exhibiting a pronounced “V”‐shaped reversible trend. This behavior indicates that tensile deformation induces enhanced axial orientation of the semicrystalline and density‐fluctuation structures within the sheath, which largely recover after stress release. Such reversible structural evolution directly reflects the dual‐network–mediated reorganization mechanism within the sheath. In contrast, the PDMS/CNT sample exhibits only minor fluctuations in within the same strain window, suggesting the absence of detectable orientation rearrangement at the nanoscale. This comparison further confirms that the reversible anisotropic evolution observed during cyclic stretching predominantly originates from the dual‐network sheath rather than the conductive core.

Figure 3F schematically illustrates the distinct deformation mechanisms of single‐ and dual‐network sheaths. In the single‐network configuration, tensile loading induces localized yielding within the PVDF sheath, leading to stress concentration and irreversible structural damage. In contrast, the dual‐network sheath forms a cooperative load‐bearing architecture, where the secondary network dissipates and redistributes stress during deformation. This mechanism suppresses localized instability and enables cooperative, reversible deformation, thereby mechanically stabilizing the compliant conductive core [46]. To further visualize this behavior, finite element simulations were performed for both SCFs and D^2^CFs during the tensile loading–unloading process (Figure S12). During unloading, the SCFs exhibit spatially heterogeneous residual stress and distinct localized stress concentrations, whereas the stress distribution in the D^2^CFs evolves more uniformly, without pronounced high‐stress regions. These results support the role of the dual‐network sheath in promoting stress redistribution and more homogeneous mechanical recovery. Consequently, deformation in the D^2^CFs is more uniformly distributed and largely reversible throughout the fiber. A comparative analysis with previously reported dry‐state porous fibers further highlights the mechanical advantages of the D^2^CFs [47, 48, 49, 50, 51, 52, 53, 54, 55]. Conventional porous fiber systems typically exhibit a trade‐off between Young's modulus and fracture strain, manifesting as either high stiffness with limited extensibility or high extensibility with insufficient mechanical robustness. This structural synergy effectively alleviates the traditional modulus–ductility trade‐off. These results indicate that the dual‐network design enhances not only nanoscale orientation regulation, as reflected by the reversible evolution of *B*_Ψ_, but also macroscopic mechanical reliability. As shown in Figure 3H, the D^2^CFs sustain a suspended load of 100 g without structural failure, demonstrating the balanced integration of stretchability and load‐bearing capacity. Introducing PEGDA as a secondary network into the PVDF sheath reprograms the deformation pathway of the fiber. The dual‐network architecture suppresses localized yielding in single‐network sheaths and promotes cooperative, reversible strain redistribution during deformation. This mechanically stabilized sheath provides the structural basis for the stable electromechanical performance discussed in the following sections.

### 2.3 Stretchable Conduction and Interpretable Resistance Dynamics

**FIGURE 4:**
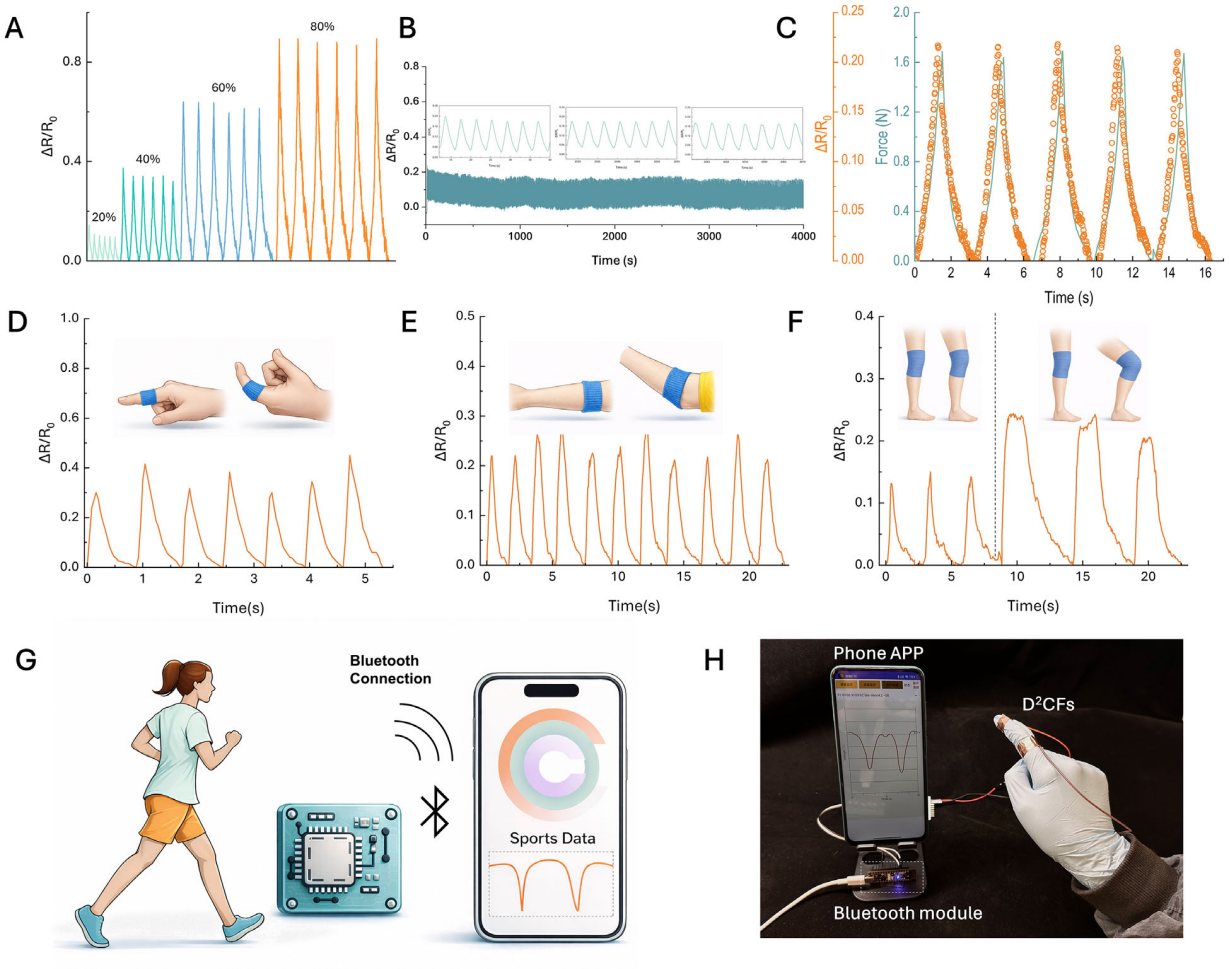
Electromechanical sensing performance and wearable demonstrations of D^2^CFs. (A) Cyclic strain‐resistive responses of D^2^CFs under different applied strains (20%, 40%, 60%, and 80%). (B) Long‐term cyclic stability of the resistance response at moderate strain, with inset showing representative signal profiles. (C) The resistance varies linearly with strain and load. (D) Resistance response of a fiber mounted on a finger during repeated bending. (E) Resistance signals recorded from elbow motion under cyclic flexion. (F) Resistance response during knee bending with distinguishable deformation amplitudes. (G) Schematic illustration of wireless transmission of motion data via Bluetooth to a mobile device. (H) Photograph of real‐time signal acquisition and display using a smartphone application.

Beyond their mechanical robustness, the coaxial fibers exhibit stable electrical behavior under strain. We first assess the static electrical stability of the D^2^CFs by continuously monitoring the resistance of a single fiber for ∼40 min under unloaded conditions. As shown in Figure S13, the resistance remains essentially constant throughout the measurement period, with no discernible baseline drift or stochastic fluctuation. This result confirms the intrinsic time stability of the PDMS/CNT conductive network under static conditions and provides a reliable electrical baseline for subsequent dynamic deformation tests. In all subsequent electrical measurements, the normalized resistance change (ΔR/R_0_) was adopted to eliminate the influence of sample‐to‐sample variation in initial resistance. Figure 4A presents the cyclic resistance response of D^2^CFs under tensile strains of 20%, 40%, 60%, and 80%. At each strain level, the fiber exhibits clear and reproducible resistance change, with signal amplitude increasing progressively with applied strain. Under moderate strain conditions (40%), extended cyclic testing was performed. Even after 1000 consecutive loading–unloading cycles, the resistance response remains highly consistent, with negligible change in peak amplitude or waveform profile (Figure 4B). The baseline drift, calculated as (= *R*_*cycle*1000_  − *R_initial_*/*R_initial_* × 100%)was only −6.54%, indicating a slight decrease in the baseline resistance and good long‐term electrical stability. This stability indicates that the conductive network does not undergo significant fracture, slippage, or irreversible reconstruction during repeated deformation within this strain regime. Statistical analysis of the resistance response under varying strain amplitudes reveals an approximately linear relationship between normalized resistance change and applied strain within the 0%–100% strain range (Figure 4C). Based on this linear regime, the calculated gauge factor (GF) is approximately 1.24 (Figure S14). Although not designed for ultra‐high sensitivity, this GF reflects a balanced electromechanical performance in which signal stability, repeatability, and long‐term reliability are prioritized. D^2^CFs also demonstrate rapid electrical responsiveness to dynamic mechanical perturbation. As shown in Figure S15, the D^2^CF exhibited a rapid resistance response of approximately 300 ms under a step‐like tensile deformation. This rapid electromechanical coupling supports potential applications in real‐time human motion monitoring and interactive systems. To further evaluate the dynamic electromechanical behavior, the electrical hysteresis of the D^2^CFs and SCFs was examined under 50% tensile strain at different stretching speeds (Figure S16). For both fibers, the electrical hysteresis decreased with decreasing stretching speed, reflecting the rate‐dependent relaxation of the polymer matrix and reconstruction of the CNT conductive network. At lower stretching speeds, the conductive pathways had sufficient time to rearrange and recover during unloading, thereby reducing the difference between the loading and unloading responses. Notably, the D^2^CFs exhibited lower hysteresis than the SCFs, particularly at higher stretching speeds, indicating that the additional network effectively restricts irreversible CNT slippage and facilitates conductive‐network recovery.

During the fast‐loading tensile tests described above, we observed that when a rapid loading step was followed by a constant‐strain holding period, the resistance signal did not remain strictly constant. Instead, it exhibited a time‐dependent evolution, gradually approaching a stable plateau. To elucidate the physical origin of this behavior, we systematically investigated the resistance relaxation of D^2^CFs under constant strain. Specifically, different levels of tensile strain were applied and maintained for 5 min, while the temporal evolution of resistance was recorded. As shown in Figure S17, at all tested strain amplitudes, the resistance exhibits a pronounced instantaneous increase (overshoot) immediately upon loading, followed by a gradual decrease toward a steady‐state value. This time‐dependent relaxation behavior closely resembles the stress relaxation commonly observed in viscoelastic materials.

Given that PDMS is a typical viscoelastic elastomer and the CNT conductive network is embedded within this matrix, the conductive pathways evolve with the time‐dependent rearrangement of polymer chains under applied strain. Accordingly, the observed resistance relaxation can be attributed to the viscoelastic stress relaxation of the polymer matrix. To quantitatively describe this behavior, the resistance relaxation curves at different strain levels were fitted using an empirical expression analogous to the classical Burgers model (Equation 2) [56].

(2) $$\frac{\Delta R}{R_{0}}=a+b\exp\left(\frac{-t}{\tau_{R}}\right)+ct$$

In this formulation, τ_*R*_ represents the characteristic resistance relaxation time, while a, b, and c are strain‐dependent fitting parameters satisfying b>0 and b×c<0. The model provides a good description of the experimental data, with a goodness‐of‐fit of R^2^ = 97.2%, and the extracted parameters are summarized in Table S1. These results suggest that, within a finite timescale, the resistance relaxation process can be treated as a quasi‐linear and parameterizable response. Further analysis of the fitted relaxation time τ_*R*_ as a function of applied strain reveals a monotonic decrease with increasing strain amplitude (Figure S18). At 150% strain, τ_*R*_ decreases to approximately 18 s. This trend indicates that larger deformation promotes more pronounced polymer chain rearrangement and accelerates the reconfiguration of the conductive filler network, thereby shortening the time required for the resistance to reach steady state. This relaxation behavior therefore originates from the viscoelastic response of the polymer matrix rather than stochastic instability of the conductive network.

Beyond standardized tensile tests, the strain‐sensing capability of the fiber was further evaluated under realistic human motion conditions. The D^2^CFs were attached to the surfaces of human joints to monitor bending of the finger, elbow, and knee (Figure 4D–F). In all cases, joint flexion produced stable and reproducible resistance variations that correlated with bending degree. Notably, in the knee‐bending experiment (Figure 4F), the fiber clearly distinguishes between mild and large‐amplitude motions. Small‐angle bending generates lower‐amplitude and narrower resistance peaks, whereas larger bending angles result in higher‐amplitude and broader signal responses. Furthermore, the D^2^CFs were integrated with a compact Bluetooth module to enable wireless data transmission (Figure 4G,H). The acquired resistance signals were transmitted in real time to a mobile application for waveform visualization. This integration confirms the compatibility of the fiber as a flexible sensing node within portable electronic systems, providing a practical foundation for wearable motion monitoring and interactive applications. The D^2^CFs exhibit stable strain‐resistive responses and physically interpretable resistance relaxation behavior under large deformation and cyclic loading. This conductive stability ensures reliable performance during dynamic strain monitoring.

### 2.4 Single‐Fiber Triboelectric Nanogenerator Enabled by Coaxial Architecture

The reversible mechanical response enabled by the dual‐network sheath stabilizes the conductive core under deformation, providing the structural basis for reliable signal generation and self‐powered transduction. Notably, the D^2^CF architecture inherently integrates a dielectric outer layer and a conductive inner electrode within a single fiber. This coaxial configuration allows direct construction of a single‐electrode‐mode triboelectric nanogenerator without additional electrodes or complex encapsulation, hereafter referred to as D^2^CF‐TENG.

**FIGURE 5:**
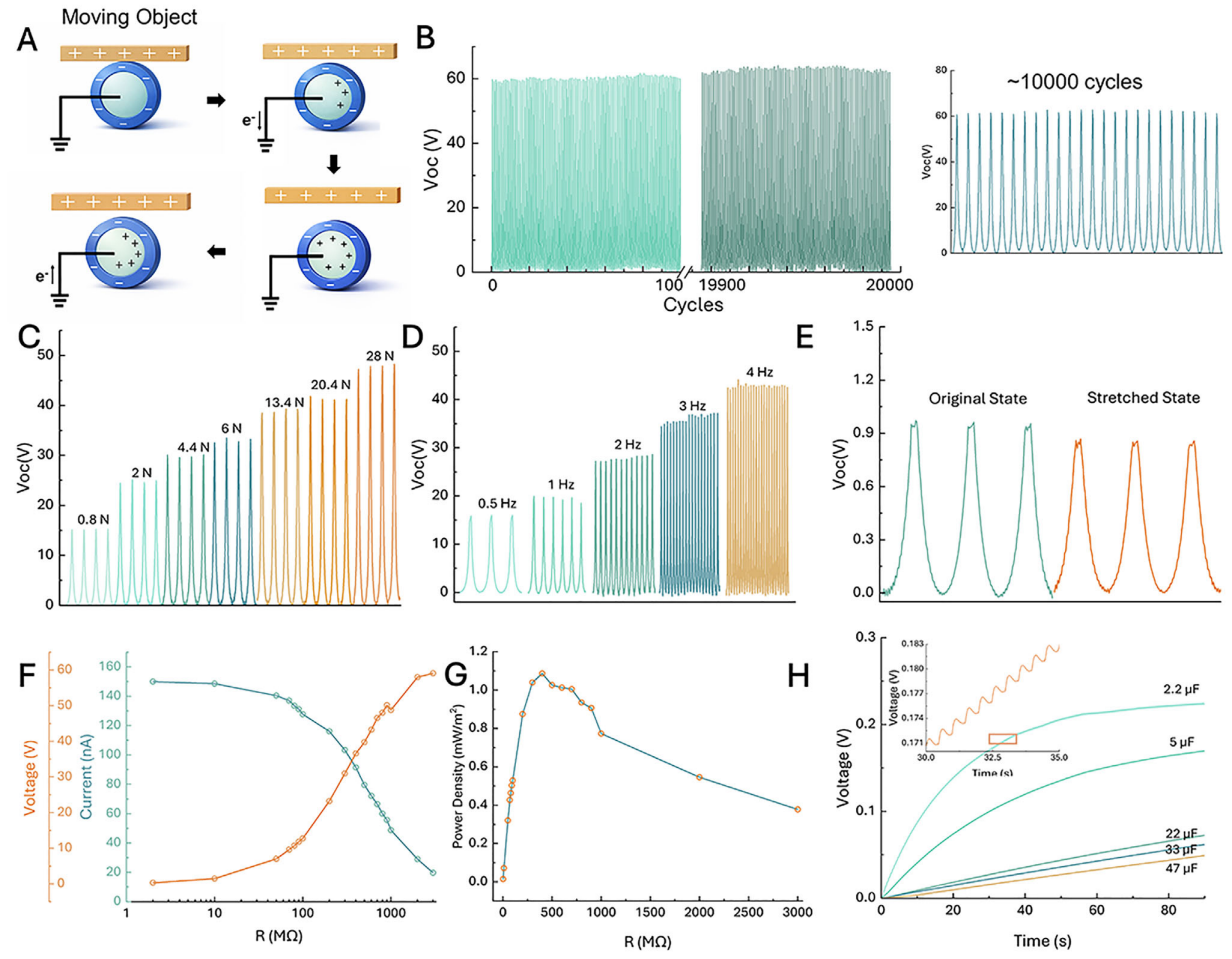
Triboelectric performance of single‐fiber D^2^CF‐TENG. (A) Schematic illustration of the working mechanism of the single‐electrode D^2^CF‐TENG during contact–separation cycles. (B) Long‐term durability test of the open‐circuit voltage (Voc) over 2 × 10^4^ contact–separation cycles, with enlarged view of representative waveforms. (C) Open‐circuit voltage outputs under different applied impact forces. (D) Open‐circuit voltage responses under varying driving frequencies. (E) Comparison of Voc signals in the original state and under 100% tensile strain. (F) Output voltage and current as a function of external load resistance. (G) Corresponding power density versus load resistance. (H) Charging curves of capacitors with different capacitances driven by D^2^CF‐TENG.

In this device configuration, the PVDF‐based sheath functions as the triboelectric dielectric layer, undergoing contact–separation interactions with an external object. The PDMS/CNT core serves as the flexible electrode and is electrically connected to ground through an external lead. As illustrated in Figure 5A, when the fiber comes into contact with a counter surface, opposite triboelectric charges are generated due to contact electrification. Upon separation, the uncompensated negative charges on the PVDF surface induce positive charge accumulation within the PDMS/CNT core, while electrons flow through the external circuit toward ground, generating a current signal. When contact is re‐established, the charge transfer direction reverses, producing a periodic output with alternating polarity. Importantly, throughout this contact–separation cycle, the conductive core remains mechanically confined and protected by the dual‐network sheath. This structural stabilization ensures that triboelectric transduction proceeds consistently under repeated mechanical excitation and large deformation, distinguishing the D^2^CF‐TENG from conventional fiber devices that may suffer from electrode instability.

Based on the above structural configuration and working mechanism, we systematically evaluated the electrical output performance of a single D^2^CF operating as a TENG. Figure 5B presents the stability test results of the device under continuous mechanical actuation. Even after 2 × 10^4^ consecutive contact–separation cycles, the open‐circuit voltage and short‐circuit current signals remain highly consistent, with no noticeable attenuation in amplitude or distortion of the waveform (Figure S19). This result indicates that the output stability of the fiber‐based TENG is not significantly affected by fatigue of the sheath or structural evolution of the conductive core, which is consistent with the mechanical and electrical stability demonstrated in the previous sections.

The influence of external driving conditions on the output performance of the D^2^CF‐TENG was further investigated. Figure 5C,D show the open‐circuit voltage responses under varying applied force and impact frequency, respectively. As the applied force increases from 0.8 to 28 N, the open‐circuit voltage exhibits a monotonic increase. Similarly, increasing the excitation frequency from 0.5 to 5 Hz results in enhanced voltage output. The force‐dependent enhancement can be attributed to more intimate contact and larger effective deformation between the fiber and the counter surface, leading to increased surface charge generation during each contact event. With respect to frequency, while the total transferred charge per cycle remains primarily determined by contact conditions, higher excitation frequency increases the rate of charge separation and transfer within a given time window. Consequently, the short‐circuit current amplitude increases with frequency, and the voltage signal exhibits enhanced peak response under dynamic excitation. The corresponding short‐circuit current trends are shown in Figures S20 and S21.

The D^2^CF‐TENG maintains stable triboelectric output even under large tensile deformation. As shown in Figure 5E, stretching the fiber to 100% strain results in negligible change in open‐circuit voltage compared with the undeformed state. This strain‐insensitive behavior indicates that the contact electrification process remains largely preserved during elongation. The dual‐network sheath maintains dielectric layer integrity, while the coaxial architecture ensures continuous electrical conduction in the PDMS/CNT core, enabling reliable triboelectric transduction under substantial deformation. To further evaluate the practical energy delivery capability of the D^2^CF‐TENG, its output characteristics were measured under varying external load resistances. As shown in Figure 5F, when the load resistance increases from 2 MΩ to 3 GΩ, the output current gradually decreases, while the output voltage correspondingly increases, consistent with typical TENG behavior. The instantaneous power density, calculated from P  = U^2^ /R, reaches a maximum value of 1.1 mW m^−^^2^ (Figure 5G). The power density was normalized based on the projected contact area of the fiber under vertical impact conditions. In addition, the charging capability of the D^2^CF‐TENG was investigated using capacitors with different capacitances (Figure 5H). Even as the capacitance increases from 2.2 to 47 µF, the device is able to charge the capacitors within a reasonable timeframe, demonstrating its potential to power low‐consumption electronic components. The D^2^CF‐TENG achieves stable and repeatable triboelectric transduction at the single‐fiber scale through its integrated core–sheath architecture. This structural stability enables reliable operation under large deformation and prolonged cyclic excitation.

### 2.5 From Single Fibers to Deployable Textile Human–Machine Interfaces

**FIGURE 6:**
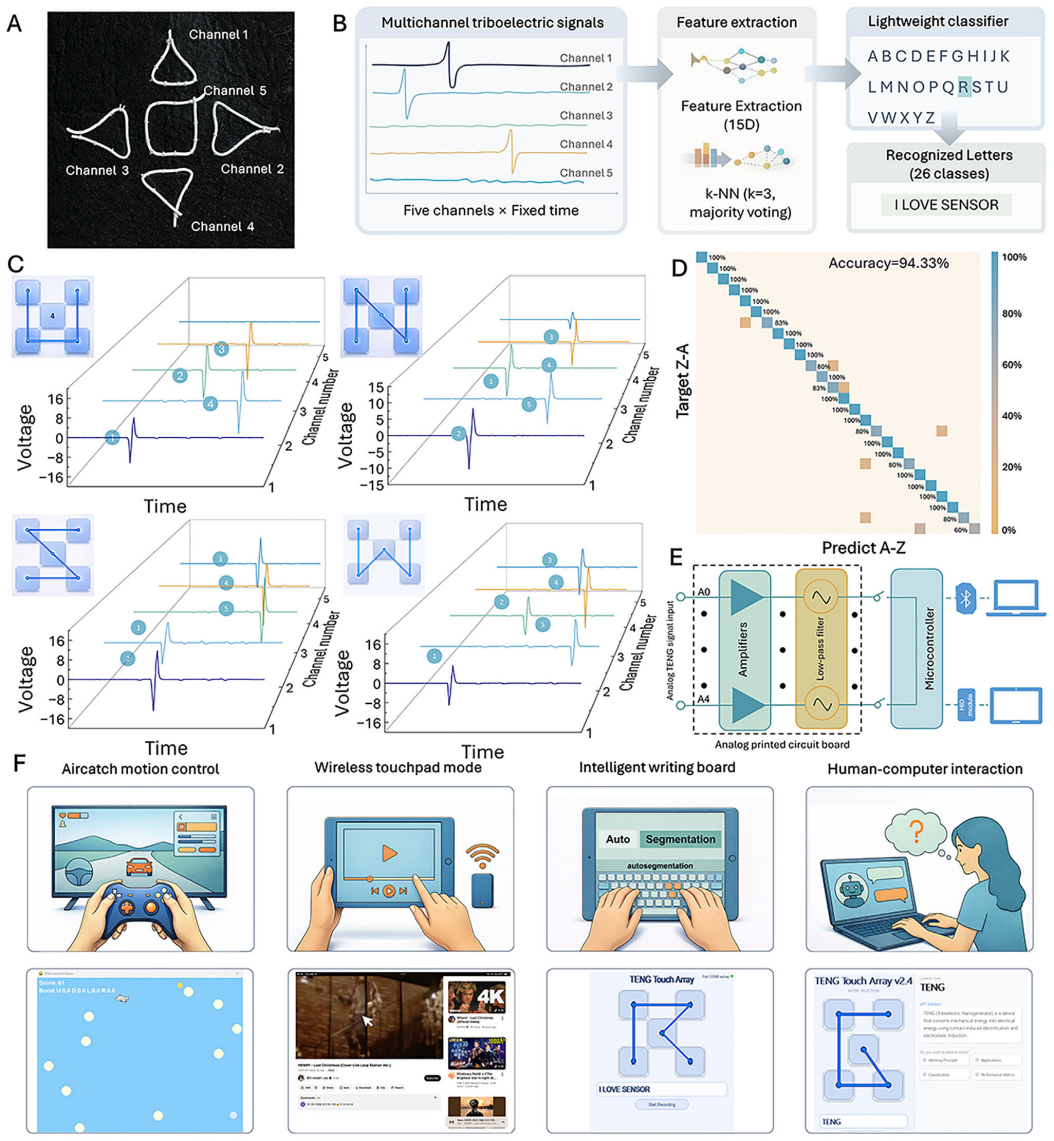
Multichannel fiber‐based human–machine interface system based on D^2^CF‐TENG. (A) Photograph of a five‐channel D^2^CF‐TENG array integrated onto textile via embroidery. (B) Signal processing pipeline including multichannel triboelectric signals, 15D feature extraction, and lightweight k‐NN classification for 26‐letter recognition. (C) Representative multichannel voltage patterns corresponding to different letter inputs and channel activation sequences. (D) Confusion matrix of 26‐letter recognition with an overall accuracy of 94.33%. (E) Circuit architecture and wireless transmission framework integrating signal conditioning, microcontroller unit (MCU), and Bluetooth HID module for real‐time communication. (F) Demonstrated application scenarios including air‐catch motion control, wireless touchpad mode, intelligent writing board, and human–computer interaction.

The coaxial fiber can also be integrated into textile platforms using conventional fabrication methods, enabling meter‐scalable and deployable human–machine interaction interfaces. Without requiring complex encapsulation or external power modules, the fiber‐based system enables multichannel input, stable signal decoding, and real‐time interaction. To construct a multichannel interactive interface, individual PVDF/PEGDA@PDMS/CNT coaxial fibers were integrated into a textile substrate via embroidery, forming a five‐channel array (Figure 6A). Each fiber unit is mechanically separated and electrically routed independently, minimizing inter‐channel interference. Under identical external stimuli, all channels produce stable and reproducible triboelectric signals with low baseline noise, indicating that the single‐fiber TENG retains output consistency after textile integration. This structural integrity provides a reliable basis for multichannel signal acquisition. Based on this array configuration, a front‐end acquisition and processing system was developed. The triboelectric signals from each channel were first conditioned through a signal‐shaping and amplitude‐limiting circuit, then synchronously sampled and transmitted to a microcontroller unit (MCU) for real‐time processing. The system supports both direct wired communication with a computer and wireless transmission via a Bluetooth module to portable devices such as tablets, enabling seamless integration with IoT platforms. Importantly, as the sensing mechanism relies on triboelectric transduction, the signal generation process itself does not require an external power supply, further simplifying system deployment.

At the signal‐processing level, a lightweight and deployable decoding strategy was adopted to avoid excessive computational overhead. For each input event, three time‐domain features were extracted from the triboelectric voltage signal of each of the five channels: (i) maximum absolute amplitude, (ii) absolute voltage integral, and (iii) the number of peaks exceeding an adaptive threshold. Each event was thus represented as a 15D feature vector. Based on these feature vectors, a k‐nearest neighbors classifier (k‐NN, k = 3) was trained to recognize different input patterns. The model was implemented in Python using the scikit‐learn library and does not rely on deep learning or large‐scale computation, making it suitable for embedded and real‐time applications (Figure 6B).

Using the multichannel input and decoding framework described above, full representation and recognition of 26 English letters were achieved (Figure S18). Each letter corresponds to a distinct channel activation sequence and temporal trajectory, enabling relatively high information density within a limited number of channels. Representative triboelectric signal profiles for the letters “U”, “N”, “S”, and “W” are shown in Figure 6C, where clear differences in temporal structure and channel activation patterns can be observed. The confusion matrix for all letter inputs is presented in Figure 6D, yielding an overall recognition accuracy of 94.33%. The dataset contained 520 input samples, comprising 26 letters with 20 repetitions for each letter. Letter recognition was based on predefined sequences of activated fiber channels after the corresponding signals exceeded the detection threshold. Importantly, this high recognition accuracy is not solely attributed to the classification algorithm but is fundamentally supported by the signal stability and reproducibility of the dual‐network fiber. As demonstrated in Sections 2.2 and 2.3, the mechanically reprogrammed sheath enables reversible deformation and suppresses structural fatigue, thereby minimizing baseline drift and signal distortion during repeated operation. This structural consistency reduces feature variability across cycles, allowing accurate decoding to be achieved using a lightweight k‐NN classifier without complex model architectures.

Figure 6E illustrates the electronic circuitry and signal transmission architecture of the integrated system. The triboelectric signals are first conditioned through the front‐end signal processing module and then transmitted either via wired communication to a computer or wirelessly through a Bluetooth HID module to portable devices such as a tablet (e.g., iPad). Through this configuration, the TENG fiber array is interfaced with IoT‐compatible platforms, enabling wireless interactive control based on self‐generated triboelectric signals. This approach provides a flexible alternative for integrating textile‐based electronics into smart control environments. Based on the hardware and decoding framework described above, multiple interaction scenarios were implemented using the fiber‐based TENG array. Figure 6F presents representative interface demonstrations, while the overall functional architecture is summarized in Figure S23. By selecting different channel activation patterns, users can switch between functional modes. In certain modes, triboelectric input signals are mapped via serial communication to directional control commands for real‐time manipulation of on‐screen objects. In other modes, the signals are translated through the Bluetooth HID protocol into mouse or touch‐control commands, enabling wireless interaction with a tablet interface. Additionally, the system supports real‐time display of recognized letters and words, facilitating text input and communication.

Beyond individual letter recognition, an automatic segmentation and sequence parsing mechanism was introduced to enable continuous character input to be converted into complete words. Specifically, the system first buffers the recognized character sequence as continuous text (raw_text). A lightweight dictionary‐based longest‐match parsing algorithm is then applied to segment the sequence. The algorithm scans the text in a case‐insensitive manner and prioritizes the longest valid word match at each position. In case no dictionary entry is matched, the parser advances character by character. In this manner, word boundaries are automatically inferred and inserted without requiring explicit space input, enabling a seamless transition from character‐level entry to word‐level expression. This mechanism enhances interaction continuity while maintaining low computational complexity.

Furthermore, the fiber‐level input platform can be interfaced with conversational information retrieval systems to extend functionality from physical input to semantic feedback. In the demonstration scenario, user‐entered letters or words generated by the multichannel TENG array are transmitted as query keywords to a backend language model, and the returned explanatory text is displayed in real time on the user interface. Importantly, the language model functions solely as an information response module and does not participate in triboelectric signal acquisition, preprocessing, or decoding. The core interaction pipeline therefore remains governed by the fiber‐based hardware and lightweight decoding framework. A detailed demonstration of the interaction process is provided in Video S1. A single PVDF/PEGDA@PDMS/CNT coaxial fiber can serve as the minimal functional unit for constructing a fiber‐based human–machine interaction interface. Through textile‐level assembly, multichannel signal acquisition, and lightweight decoding strategies, the system enables real‐time human–machine interaction with high recognition accuracy using self‐generated triboelectric signals. This capability allows D^2^CFs to function not only as sensing elements but also as active interaction units for direct information input and system‐level integration.

## 3 Conclusion

In this work, we establish a meter‐scalable design and manufacturing method for fiber‐level multiphysics integration through a coaxial wet‐spinning strategy. By introducing a core–sheath functional decoupling design and incorporating a PEGDA secondary network within the PVDF sheath, elastomeric conductors that are typically incompatible with aqueous coagulation bath wet spinning can be continuously integrated into fiber fabrication. The resulting dual‐network sheath stabilizes the conductive cores and enables reversible deformation under large strain, leading to reliable electromechanical responses and stable triboelectric output during repeated mechanical loading. Owing to this structural robustness, the fibers can be directly assembled into multichannel embroidered arrays to serve as self‐powered sensing elements in human–machine interaction systems. By combining meter‐scalable fiber manufacturing with lightweight signal processing algorithms, the resulting textile interface enables tactile sensing and character‐level input with high recognition accuracy. Overall, this work demonstrates a coherent pathway from structural materials design and meter‐scalable fiber manufacturing to textile‐level electronic systems. The proposed coaxial fiber architecture provides a versatile platform for integrating soft conductive materials, multiphysics functionality, and interactive sensing within a single fiber unit, offering new opportunities for next‐generation wearable electronics and intelligent textiles.

## 4 Methods

### 4.1 Preparation of Sheath Spinning Solution (PVDF‐HFP/DMF)

PVDF‐HFP (Mw ≈ 400 000, Arkema Kynar Flex 2801, China) was dissolved in N,N‐dimethylformamide (DMF, analytical grade) to obtain a 20 wt.% solution. The mixture was magnetically stirred for 4 h at room temperature until a transparent and homogeneous solution was obtained.

### 4.2 Preparation of Core Spinning Solution (PDMS/CNT Prepolymer)

0.25 g of carbon nanotubes (CNT, TNM1, Time Nano, China) were dispersed in 20 mL of hexane (Honeywell, USA) and mixed with 9 g PDMS Part A (SYLGARD 184, DowSil, USA). The mixture was ultrasonicated for 30 min in an ice‐water bath to ensure uniform dispersion. The resulting PDMS/CNT/hexane mixture was magnetically stirred at low speed under ambient conditions to allow complete evaporation of hexane. After solvent evaporation, PDMS Part B was added to the PDMS/CNT mixture at a weight ratio of 10:1 (Part A: Part B) and thoroughly mixed. The core spinning solution was used immediately after preparation to prevent CNT aggregation, which could affect viscosity and electrical conductivity.

### 4.3 Coaxial Wet‐Spinning Process

The core and sheath spinning solutions were separately loaded into 10 mL syringes mounted on syringe pumps. A 15G/20G coaxial needle was employed, and deionized water was used as the coagulation bath. The sheath solution was first extruded at 10 mL h^−^^1^ to establish a stable sheath jet. Subsequently, the core solution was introduced at 12 mL h^−^^1^ to form the coaxial structure within the water bath. Core flow rates ranging from 12 to 15 mL h^−^^1^ produced stable fibers with satisfactory morphology; therefore, 12 mL h^−^^1^ was selected for subsequent fabrication. After spinning, the fibers were immersed in deionized water to ensure complete solvent exchange between DMF and water. The fibers were then transferred to deionized water at 70°C for 4 h to fully cure the PDMS core. Finally, the fibers were removed and dried overnight at room temperature.

### 4.4 Preparation of Sheath Spinning Solution (PVDF‐HFP/PEGDA/DMF)

PEGDA (Mn ≈ 250, Sigma–Aldrich, USA) and Omnirad 184 (photoinitiator, BASF, Germany) were added to the PVDF‐HFP/DMF solution described above. The mass ratio was: PVDF‐HFP: Omnirad 184: PEGDA = 100:2:4. The mixture was magnetically stirred for 1 h to obtain a homogeneous sheath spinning solution. The core solution preparation and coaxial wet‐spinning process were identical to those described above. After drying, the fibers were exposed to UV light (365 nm, 40 W, UV density = 1500 uw/cm^2^) for 10 min to initiate polymerization of PEGDA within the sheath, forming the dual‐network structure.

### 4.5 Characterization

Fiber morphology was examined using scanning electron microscopy (SEM, TM4000 Plus, Hitachi, Japan). For cross‐sectional observation, fibers were fractured after immersion in liquid nitrogen. Fourier Transform Infrared (FT‐IR) spectroscopy was carried out using a Spectrum 100 (PerkinElmer, UK) over a wavenumber range of 650–4000 cm^−^^1^ with a resolution of 16 cm^−^^1^. Raman spectra were recorded using an inVia Raman microscope (Renishaw, UK) equipped with a 532 nm laser and a 2400 lines mm^−^^1^ grating. Mechanical properties were measured using a universal testing machine (Instron 5966, Norwood, USA). The initial gauge length was set to 10 mm. The tensile speed was 10 mm min^−^^1^, and cyclic tensile tests were conducted at 20 mm min^−^^1^. The stress retention was calculated as the ratio of the maximum stress at the 500th cycle to that at the first cycle. In situ small‐angle x‐ray scattering (SAXS) experiments were conducted at beamline BL16B1 of the Shanghai Synchrotron Radiation Facility (SSRF). Samples were mounted on a tensile stage (TST 350) and subjected to cyclic tensile strain up to 60%. Twenty scattering patterns were collected during a single loading–unloading cycle. Resistance variation was measured using a digital multimeter (Keithley DMM6500) in two‐wire mode with a time resolution of 2 ms. Mechanical loading was applied using a force gauge (Mark‐10). Triboelectric output performance was evaluated under ambient conditions at room temperature and a relative humidity of 30%–50%. A commercial linear motor system (Zaber LSQ450B‐T3A) was used to generate periodic contact–separation motion, with nitrile rubber serving as the contact material. The active fiber length and separation distance were fixed at 3 and 10 mm, respectively. The displacement and operating frequency were controlled using Zaber Console software. The applied impact force was monitored in real time using a force tester (Mark‐10 M5‐200), while the open‐circuit voltage was measured using an electrometer (Keithley 6514). All electrical signals were recorded and analyzed in real time using a custom LabVIEW program. The instantaneous output power delivered to the external load was calculated according to (*P*  = *V* ^2^ /*R*), where (*V*) is the voltage measured across the load resistance (*R*). The corresponding power density was calculated as (*P_density_* = P/*A* ), where (*A*) is the projected area of the tested fiber. The projected area used for normalization was (0.003375m^2^).

## Author Contributions

**Shengjie Ling**: conceptualization, methodology, supervision, formal analysis, Writing – review and editing. **Ming Li**: methodology, formal analysis, writing – review and editing, investigation. **Chun‐Hui Wang**: conceptualization, formal analysis, supervision, writing – review and editing. **Xinyi Cao**: conceptualization, methodology, software, data curation, investigation, validation, formal analysis, writing – original draft. **Shuhua Peng**: conceptualization, validation, formal analysis, supervision, resources, project administration, funding acquisition, writing – review and editing. **Chao Ye**: conceptualization, methodology, software, writing – review and editing. **Zhao Sha**: conceptualization, methodology, formal analysis, resources, writing – review and editing.

## Conflicts of Interest

The authors declare no conflicts of interest.

## Supporting information




**Supporting File 1**: advs77580‐sup‐0001‐SuppMat.docx.

**Supporting File 2**: advs77580‐sup‐0002‐VideoS1.mp4.

## Data Availability

The data that support the findings of this study are available from the corresponding author upon reasonable request.
