# Supplementary material for: Mechanically Reprogrammed Coaxial Fibers for Multiphysics Transduction and Human‐Machine Interfaces
Source: Adv Sci (Weinh). 2026 Sep 6:e77580. Online ahead of print. doi: 10.1002/advs.77580 (PMC13547702; doi:10.1002/advs.77580)
Supplement: Supplementary file 1 [file ADVS-9999-e77580-s001.docx]

Supporting Information

Mechanically Reprogrammed Coaxial Fibers for Multiphysics Transduction and Human-Machine Interfaces

Xinyi Cao, Chao Ye*, Shengjie Ling*, Ming Li, Zhao Sha*, Chun Wang, Shuhua Peng*

Supporting information contains:

Figure S1-S23

Table S1

Note S1

Movie S1

**Figure S1.** FTIR spectra of PVDF, PVDF/PEGDA before crosslinking, and UV-crosslinked PVDF/PEGDA.

**Figure S2.** SEM image showing the dispersion of CNTs within the PDMS matrix.

**Figure S3.** Raman spectra of pure CNTs and the PDMS/CNT composite.

**Figure S4.** Photograph of D²CFs embroidered onto a fabric substrate forming the pattern “UNSW” and a flower.

**Figure S5.** Cyclic tensile stress–strain curves of the standalone PDMS/CNT elastomeric conductor over 500 loading–unloading cycles.

**Figure S6.** Stepwise loading–unloading tensile curves of the PDMS/CNT elastomeric conductor under progressively increasing strain from 20% to 200%.

**Figure S7.** Statistical comparison of the Young’s modulus and elongation at break of D2CFs and SCFs.

**Figure S8.** Evolution of mechanical hysteresis of D²CFs and SCFs during 500 tensile–release cycles.

**Figure S9.** Stepwise loading–unloading tensile curves of SCFs with strain progressively increased from 20% to 100%.

**Figure S10.** Stepwise loading–unloading tensile curves of D²CFs with strain progressively increased from 20% to 100%.

**Figure S11.** In situ SAXS patterns collected during a complete loading–unloading cycle. (A) D²CFs, showing the full set of 20 SAXS patterns recorded within one deformation cycle. (B) PDMS/CNT composite, showing the corresponding 20 SAXS patterns collected under the same testing conditions.

**Figure S12.** Finite element simulation of the axial stress distribution in SCFs and D²CFs during tensile loading and unloading.

**Figure S13.** Time-dependent resistance of D²CFs measured over ~40 min under static conditions.

**Figure S14.** Relative resistance change (ΔR/R₀) of D²CFs as a function of applied strain for gauge factor (GF) calculation.

**Figure S15.** Rapid resistance response of D²CFs under fast loading–unloading deformation.

**Figure S16.** Comparison of the electrical hysteresis of D²CFs and SCFs at different tensile rates.

**Figure S17.** Resistance relaxation behavior of D²CFs under constant strain at different strain levels, together with fitting of the ΔR/R₀–time curves.

**Figure S18.** Strain-dependent relaxation time $\tau_{R}$ extracted from the fitting of resistance relaxation curves of D²CFs.

**Figure S19.** Short-circuit current of the D²CF-TENG measured during 20,000 continuous contact–separation cycles.

**Figure S20.** Short-circuit current of the D²CF-TENG under different forces.

**Figure S21.** Short-circuit current of the D²CF-TENG under different driving frequencies.

**Figure S22.** Demonstration of the 26 English letters and their corresponding triboelectric signal patterns generated by the multi-channel D²CF-TENG system.

**Figure S23** Schematic illustration of the four functional frameworks of the human–machine interaction system.

**Table S1.** Fitting parameters extracted from the resistance relaxation curves of D²CFs at different strain levels.

| Strain | a | b | c |
| --- | --- | --- | --- |
| 20%  50%  100%  150% | -0.101683  -0.179891  -0.264933  -0.436325 | 0.053682  0.086498  0.108879  0.162403 | -1.036744e-04  -1.702109e-04  -1.737315e-04  -2.429054e-04 |

**Note S1.** Quantitative analysis of SAXS patterns and calculation procedure.

To quantitatively evaluate the orientation state of the anisotropic scatterers, two-dimensional SAXS patterns were azimuthally integrated. The orientation parameter $B_{obs}$ was calculated according to:

$$B_{obs}(s)=\frac{\int I\left( s,\varphi\right)d\varphi}{I(s,\frac{\pi}{2})}$$

Where $I\left( s,\varphi\right)$ is the scattering intensity at a given at a given scattering vector $s$ and azimuthal angle $\varphi$. The denominator $I\left( s,\varphi\right)$ corresponds to the intensity measured along the equatorial direction.

For each fixed scattering vector $s$, a unique $B_{obs}$ value is obtained through azimuthal integration of the intensity distribution. Thus, $B_{obs}$ reflects the angular broadening of the scattering profile and quantitatively describes the orientation distribution of the scatterers.

The experimentally observed slope parameter$B_{obs}$ follows:

$$sB_{obs}\left( s \right)=sB_{\Psi}+\frac{1}{L}$$

where $B_{\Psi}$ represents the contribution from the orientation distribution function, and $L$ denotes the mean length of the scattering objects.
